# Opioid/Dopamine Receptor Binding Studies, NMR and Molecular Dynamics Simulation of LENART01 Chimera, an Opioid-Bombesin-like Peptide

**DOI:** 10.3390/molecules29010272

**Published:** 2024-01-04

**Authors:** Pawel Serafin, Łukasz Szeleszczuk, Igor Zhukov, Edina Szűcs, Dávid Gombos, Azzurra Stefanucci, Adriano Mollica, Dariusz Maciej Pisklak, Patrycja Kleczkowska

**Affiliations:** 1 Department of Military Health Service, Ministry of National Defence of the Republic of Poland, Niepodleglosci 211 Street, 00-911 Warsaw, Poland; pawelserafin1@wp.pl; 2 Department of Organic and Physical Chemistry, Faculty of Pharmacy, Medical University of Warsaw, Banacha 1 Street, 02-093 Warsaw, Poland; lukasz.szeleszczuk@wum.edu.pl (Ł.S.); dariusz.pisklak@wum.edu.pl (D.M.P.); 3 Institute of Biochemistry and Biophysics, Polish Academy of Sciences, Pawińskiego 5a Street, 02-106 Warsaw, Poland; igor@ibb.waw.pl; 4 Institute of Biochemistry, Biological Research Centre, Hungarian Research Network, Temesvári krt. 62, H-6726 Szeged, Hungary; szucs.edina@brc.hu (E.S.); gombos.david@gmail.com (D.G.); 5 Doctoral School of Theoretical Medicine, Faculty of Medicine, University of Szeged, Dugonics Square 13, H-6720 Szeged, Hungary; 6 Department of Pharmacy, G. d’Annunzio University of Chieti-Pescara, 66100 Chieti, Italy; a.stefanucci@unich.it (A.S.); a.mollica@unich.it (A.M.); 7 Maria Sklodowska-Curie Medical Academy in Warsaw, Solidarnosci 12 Street, 03-411 Warsaw, Poland

**Keywords:** receptor binding, dopamine receptors, hybrid compound, molecular docking

## Abstract

The design and development of hybrid compounds as a new class of drug candidates remains an excellent opportunity to improve the pharmacological properties of drugs (including enzymatic stability, efficacy and pharmacokinetic and pharmacodynamic profiles). In addition, considering various complex diseases and/or disorders, the conjugate chemistry approach is highly acceptable and justified. Opioids have long been recognized as the most potent analgesics and serve as the basic pharmacophore for potent hybrid compounds that may be useful in pain management. However, a risk of tolerance and physical dependence exists. Since dopamine receptors have been implicated in the aforementioned adverse effects of opioids, the construction of a hybrid with dual action at opioid and dopamine receptors is of interest. Herein, we present nuclear magnetic resonance (NMR) spectroscopy and molecular dynamics simulation results for LENART01, an opioid–ranatensin hybrid peptide. Apart from molecular docking, protein–ligand interactions were also assessed in vitro using a receptor binding assay, which proved LENART01 to be bound to mu-opioid and dopamine receptors, respectively.

## 1. Introduction

In the era of the dramatic development of multimorbidity, generating the need for the simultaneous use of many drugs and thus increasing the risk of side effects as a result of drug–drug interactions, a hybrid approach emerges as the only possible solution to the problem. Hybrid structures, combinations of two biologically/pharmacologically distinct entities (pharmacophores), have more favorable profiles than their components. Indeed, they may have superior safety or efficacy, as their potency is usually more significant than the sum of individual building blocks. In addition, the pharmacokinetics of a single drug are preserved, reducing the likelihood of drug–drug interactions [1,2]. However, these molecules have some limitations. They are usually large molecules with physicochemical properties that exceed Lipinski’s rule-of-five [3], which is particularly interesting in discovering orally bioavailable drugs.

Molecular docking approaches uncover critical elements in protein–ligand interactions, thereby enabling the development of different drug candidates that act at specific targets with a predicted binding mode. However, because either receptors or enzymes exhibit conformational flexibility [4,5], the ligand–target matching results are sometimes inaccurate and unreliable [6]. Moreover, despite presenting the structural features of two “parent” building elements, hybrid compounds may have entirely new biological activities and properties [2]. To achieve the desired effect, such structures may simultaneously hit several other targets that are not directly related to the receptors/enzymes of interest [2,7]. In this respect, determining any possible interaction, especially of hybrid structures, is of great interest to allow for a reliable discussion of the observed therapeutic and/or toxic effects.

In the present study, we presented some features of a new chimera—LENART01, which has previously shown its antimicrobial activity against different *E. coli* strains [8]. Noteworthily, the N-terminal pharmacophore of LENART01 consists of dermorphin with an amino acid sequence of YdAFGYPS. At the same time, the C-terminus was constructed from ranatensin, which was additionally heavily modified, resulting in a sequence of GHFM. Since both the opioid pharmacophore and ranatensin are known to interact with the mu-opioid receptor (MOR) and the dopamine D2 receptor (D2R), respectively [9,10,11], it was hypothesized that the biological effects elicited by the chimera could also be due to these interactions. Therefore, we demonstrate NMR studies and a molecular dynamics (MD) simulation to validate the results of classical in vitro binding studies performed for a newly developed bivalent ligand, LENART01, comprising opioid- and dopamine-receptor-related pharmacophores.

## 2. Results and Discussion

### 2.1. Multidimensional NMR Spectroscopy

**Figure 1:**
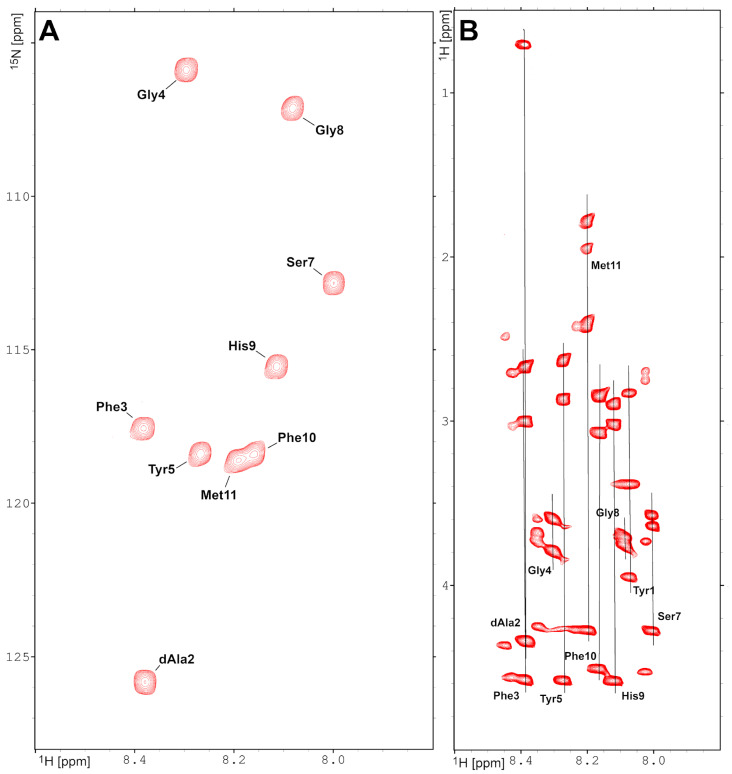
Two-dimensional NMR spectra collected for LENART01 chimeric peptide at 298 K. (**A**) Heteronuclear 1H-15N HSQC spectrum acquired on the natural abundance of the ^15^N isotope. (**B**) Fragment of the ^1^H-^1^H TOCSY spectrum collected with mixing time 80 ms. The assignment of ^1^H and ^15^N resonances is shown on the spectra.

The assignment procedure performed for the LENART01 chimeric peptide was based on the joint analysis of homonuclear (TOCSY, ROESY) and heteronuclear (^1^H-^13^C, ^1^H-^15^N HSQC) spectra acquired with a natural abundance of the ^13^C and ^15^N isotopes. Initial assignments were achieved by analysis of the heteronuclear ^1^H-^13^C HSQC spectrum (Figure S1). The application edition that distinguished signals from the CH_3_, CH_2_ and CH groups made it possible to assign most of the ^1^H and ^13^C resonances. These data were supplemented by the ^1^H-^13^C HSQC spectrum, recorded with parameters tuned to aromatic resonances (Figure S2). Finally, assignments obtained for aliphatic ^1^H were transferred to heteronuclear 1H-15N HSQC (Figure 1A) and homonuclear TOCSY (Figure 1B) spectra. As a result, practically all resonances (96%) presented in the LENART01 peptide were successfully assigned (Table S1).

A high-resolution 3D structure was elucidated based on 65 distance constraints, including 3 medium-range and 2 long-range constraints yielded from the analysis of the 2D ROESY spectrum (Figure S3), which were collected with a mixing time 400 ms. Additional restraints for φ and ψ backbone torsion angles were obtained from the analysis of ^1^H, ^13^C and ^15^N chemical shifts with the TALOSn program [12] (Figure S4). Finally, additional distance constraints for three hydrogen bonds were defined between Tyr5–His9, Pro6–Phe10 and Ser7–Met11 based on geometric criteria and were used for a refinement step of 3D structure determination.

**Figure 2:**
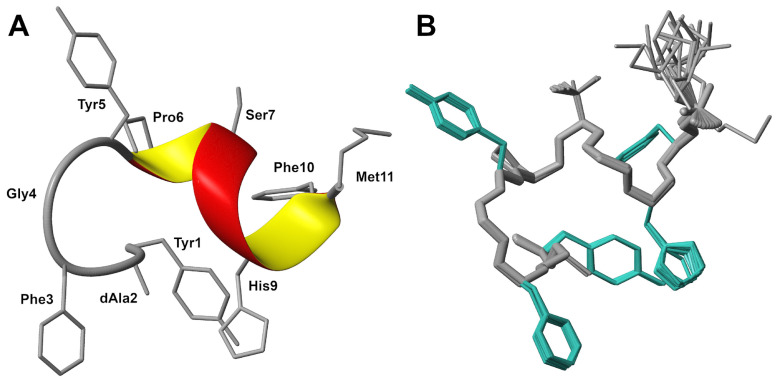
High-resolution 3D structure of the LENART01 chimera peptide in DMSO-d_6_. (**A**) Ribbon presentation of 3D structure, with a-helical conformation in the C-terminal fragment. (**B**) The ensemble of 20 low-energy structures obtained based on 230 distance constraints yielded from analysis of the ROESY experiment.

The solved 3D structure demonstrated that LENART01 in DMSO-*d_6_* was represented as a folded domain, with two turns of the α-helical structure that included the Tyr5–Met11 residues in the C-terminal fragment (Figure 2) and no outliers in backbone torsion angles (Figure S5).

The solved high-resolution 3D structure of the LENART01 peptide presented a one-turn α-helix in the C-terminal region comprising Pro6–Phe10 (Figure S6). In contrast, as supposed, the N-terminal fragment had no propensity to form an α-helical conformation, which was due to the presence of specific amino acids in the LENART01 sequence that are known for their helix-breaking properties (i.e., proline and glycine) [13].

### 2.2. Receptor Binding Affinities and Activation Efficacy of LENART01 towards Opioid and Dopamine Receptors

**Figure 3:**
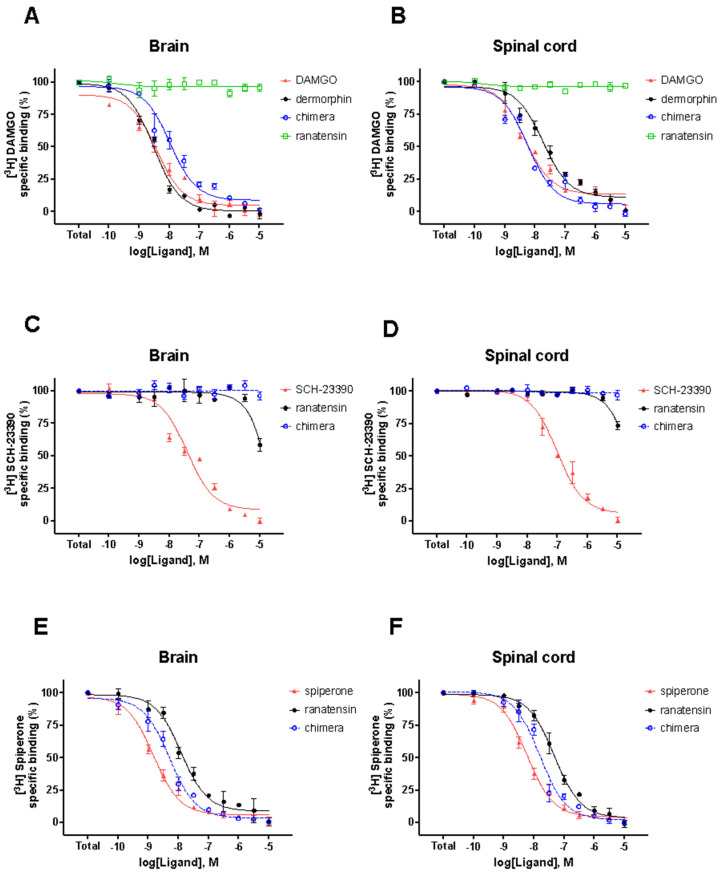
Binding affinity of LENART01 at MOR (**A**,**B**), D1R (**C**,**D**) and D2R (**E**,**F**) receptors, respectively, using [^3^H]DAMGO and [^3^H]SCH−23390 and [^3^H]Spiperone as radioligands. Results represent mean ± SEM of 3 independent experiments, each performed in duplicate.

The chimeric peptide LENART01 was examined in [^3^H]DAMGO, [^3^H]SCH−23390 and [^3^H]Spiperone homolog displacement in rat brains and spinal cord homogenates. The binding affinity of the hybrid and its intrinsic activity value were compared with dermorphin and ranatensin, its structural components. As presented, dermorphin and the chimera showed similar binding affinities (IC_50_ value) in the μ-opioid system to DAMGO, while ranatensin did not bind to MOR (Figure 3A,B). This agrees with Zhu et al. [10], who demonstrated ranatensin-induced analgesia mediated by dopaminergic receptors, as naloxone failed to antagonize the pain-relieving effect. However, in the dopaminergic system (Figure 3C–F), LENART01 showed high selectivity to D2R compared to D1R. Such results suggest that LENART01 may ultimately be devoid of the side effects of the opioid pharmacophore, which proves the overall concept of hybrid drugs as compounds with fewer side effects and reduced toxicity compared to their single-building elements [14,15,16].

For functional binding, the dose-dependent increases in the chimera were equal to the increases in dermorphin in rat brain membranes (Table 1). In contrast, in the spinal cord membrane, the maximal stimulation of the chimera was less than that of dermorphin (Table 1). The selective MOR antagonist cyprodime reversed the efficacy of the chimera, and the selective D2R antagonist risperidone brought down the stimulation of LENART01 to basal activity. The selective D1R antagonist SCH−39166 did not significantly reverse the efficacy of the chimera.

### 2.3. Molecular Docking and MM/GBSA Calculations of the Complexes Formed between LENART01 and D2R or MOR Receptors

Understanding the mechanisms of compound action and simulating the events that occur during receptor activation are now possible through experimental and computational approaches. Molecular modeling methods generate chemical action predictions using various kinds of data. Ligand-based procedures merely use information on the chemical structures of substances, whereas structure-based approaches are based on the spatial orientation of the target protein’s atoms and use docking to predict ligand fitting at the proper binding site. A 3D model of a protein–ligand complex structure can now be created using computational techniques, and atomic-level analyses of the processes underlying protein–ligand binding ability may also be performed. Most of this research is based on ligand docking, scoring function computations, molecular dynamics (MD) simulations and binding free energy (ΔG) calculations.

In order to forecast the interactions between the ligand (LENART01) and D2R or MOR receptors, this study employed in silico techniques. First, molecular docking was performed to search for the most favorable possible positions of the chimera in the complexes with either MOR or D2R, and to estimate the ligand binding affinities. Optimized ligand molecules were docked into the optimized receptor-modeled structures using the flexible docking protocol to enable a search for all probable conformations generated due to changes in residue torsion angles.

**Figure 4:**
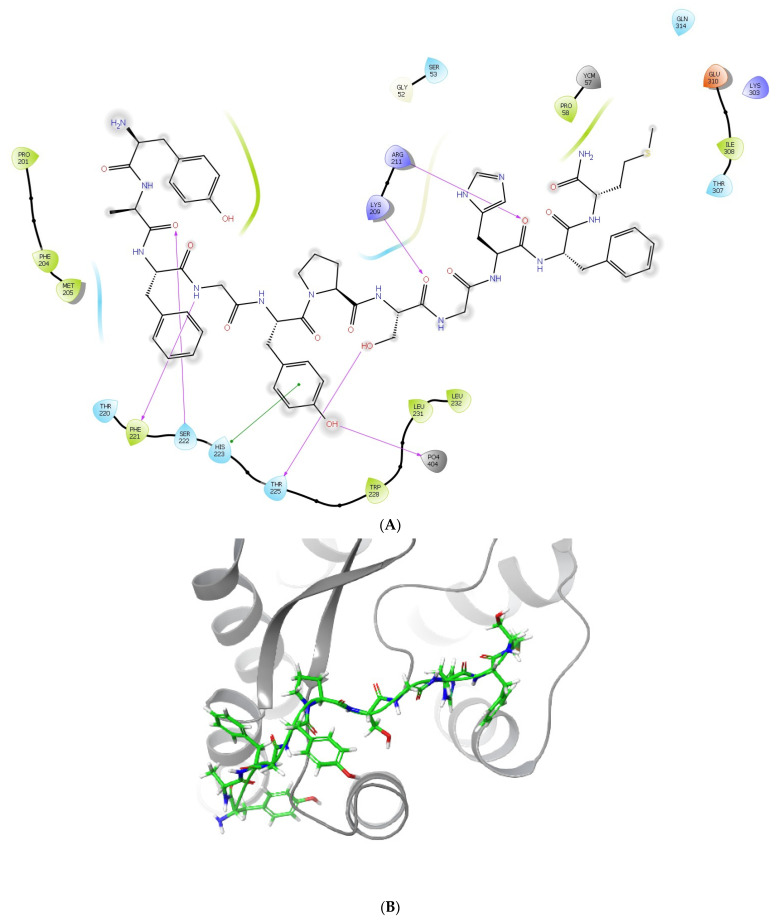

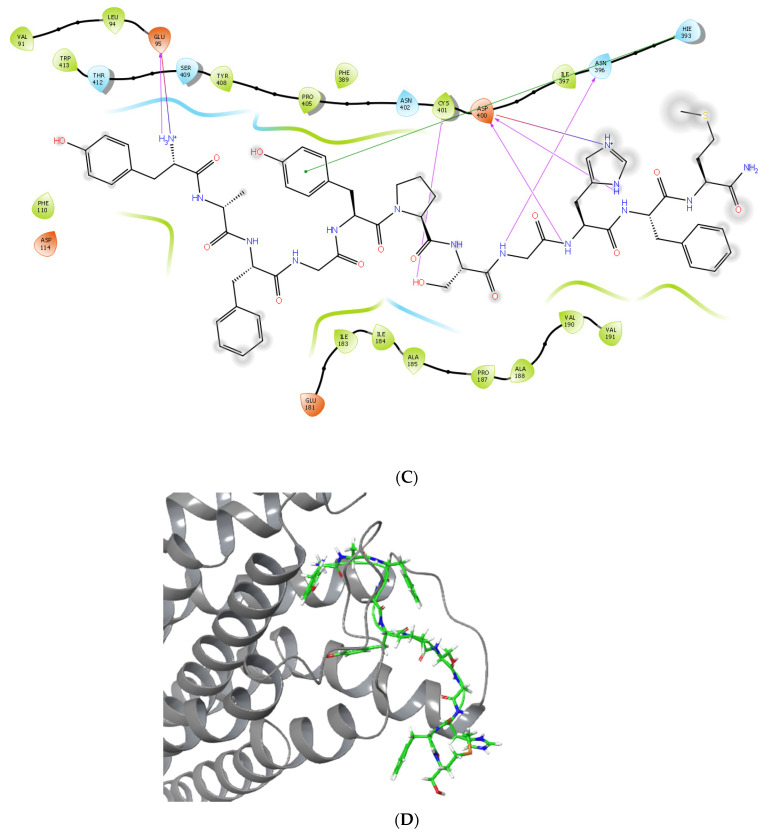
Ligand interaction diagrams (**A**,**C**) and binding site visualizations (**B**,**D**) of the complexes formed between LENART01 and MOR (**A**,**B**) and D2R (**C**,**D**), respectively.

Based on the calculations of ligand scoring function values (Glide score, Glide emodel—Table 2), we elucidated the sites that best accommodate LENART01 (Figure 4). Visualization of these complexes allowed for the identification of the residues forming interactions with the studied ligand. Using the MD trajectory outlined in Section 3, the molecular-mechanics-generalized Born and surface area (MM-GBSA) approach was used to obtain the binding free energies for each binding mode.

In both cases, namely, the complexes of the studied ligand with MOR and D2R, the negative values of the scoring functions as well as the MM/GBSA ΔG_binding_ indicated that the complexes between the chimera and the studied receptors were formed spontaneously, and they were both energetically and thermodynamically stable. However, the residues and functional groups of LENART01 employed in creating those complexes varied significantly between the structures. In the case of the complex formed between LENART01 and MOR, three carbonyl oxygen atoms were acceptors of H-bonds, forming interactions with LYS209, ARG211 and SER222. Another H-bond was formed between the hydroxyl group of LENART01′s Ser and THR225. Another interesting force was a π-cation interaction formed between the aromatic ring of the ligands Tyr and HIS223, as the same type of bonding could also be observed in the complex with D2R, this time formed with HIS393.

Multiple hydrogen bonds could also be observed in the complex formed between the studied ligand and D2R, formed with protein residues such as CYS182, ASP400 and CYS401. Despite the lower number of peptide–protein interactions in this complex, its docking scores and MM/GBSA ΔG_binding_ were found to be more negative than in the case of MOR. This may indicate a higher affinity of the ligand to this particular receptor, which agrees with the study of Raschka et al. [17], demonstrating that strong H-bonds are required for most high-affinity ligands. However, a detailed analysis of the particular contributions to the total MM/GBSA energy from different components, presented in Table 3, showed that the major differences between the complexes were in the Coulomb energy and generalized Born electrostatic solvation energy, indicating differences in the interactions stabilizing particular systems.

### 2.4. Molecular Dynamics Simulations of the Complexes Formed between LENART01 and D2R or MOR Receptors

Molecular docking methods yield ligand–receptor complexes that only record a single instant of the mutual orientation between a ligand and a target protein. In contrast, molecular dynamics (MD) simulations constitute a more computationally expensive approach that yields substantially more information. By simulating the behavior of the modeled system (such as a ligand–protein complex) over time, this method makes it possible to acquire additional ligand–protein poses. As such, more information is given as more postures are taken into account. Therefore, in order to understand the activity patterns of the LENART01 hybrid peptide, we used molecular docking and MD simulations in this work. The root-mean-square deviation of atomic positions (RMSD) and root-mean-square fluctuation (RMSF) are the most common measures of compound structural mobility [18]. RMSF is a numerical measurement similar to RMSD. Still, instead of indicating positional differences between entire structures over time, RMSF calculates individual residue flexibility, or how much a particular residue moves (fluctuates) during a simulation. In this aspect, Figures S7 and S8 show the RMSD plots obtained for Cα-atoms of MOR and D2R in the complexes formed with the chimera during a 100 ns MD simulation. It can be seen that in the case of the MOR complex, the RMSD stabilized itself at the level of 3.5 Å after c.a. 60 ns of simulations, while for the D2R complex, the plateau was obtained at the level of 4.2 Å after c.a. 45 ns of simulations. Since it is known that high RMSD values are correlated to significant instability, being related to changes within the conformation of the investigated molecule [19], the results obtained herein present protein molecules in the studied complexes to be stable from a structural point of view.

The RMSF graphs obtained for the Cα-atoms of MOR and D2R complexes generated with LENART01 during a 100 ns MD simulation are also displayed in Figure S9. The greatest relative mass difference (RMSD) per residue in both proteins was greater than 0.8 Å, and often did not surpass 4.0 Å. This suggests that the examined model of macromolecules is accurate, because no unusually highly dynamic residues were found.

The secondary structure (SS) content of the proteins under study in their complexes with LENART01 during the MD simulation is displayed in Figure S10. With the exception of the areas with high RMSF values, which were primarily associated with unstructured receptor sections, the great majority of residues maintained a stable alignment to the specific kind of SS throughout the simulation. Since the SS content in both scenarios remained constant throughout the simulation, no significant structural changes were seen.

Multiple weak, low-energy (1–5 kcal/mol) noncovalent interactions, such as H-bonds, ionic and hydrophobic forces, at short distances sufficient for bonding (usually 2.5–3.5 Å) create specific protein–ligand connections.

**Figure 5:**
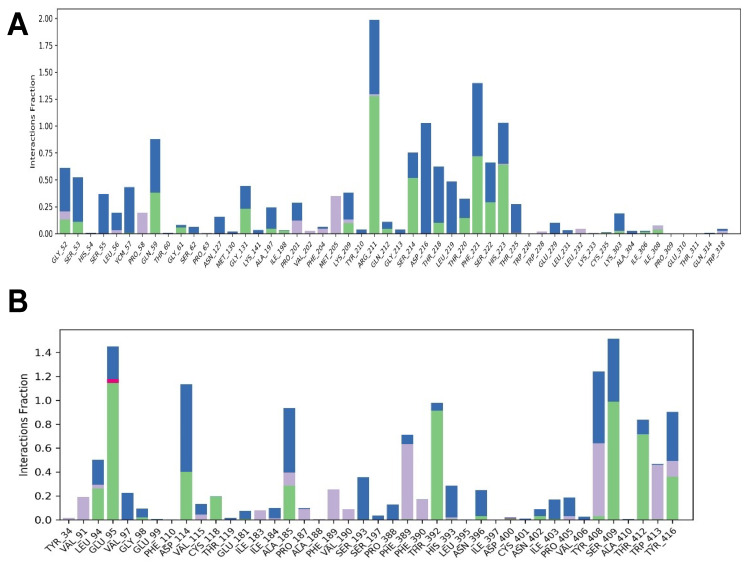
Protein–ligand interaction chart illustrating the percentage of simulation time devoted to LENART01-mediated interaction with D2R residues (**panel B**) and MOR (**panel A**). The interactions involving hydrogen bonding across a water bridge molecule are represented by the color blue. For the hydrogen bonds to the bridging water, the geometric requirements were an H-A distance of less than 2.7 Å, a D-H-A angle of more than 110°, and an H-A-X angle of more than 80°. Hydrogen bonds are indicated by a green hue and were determined by the D-H lengths and angles. An H-A distance of less than 2.8 Å, a D-H-A angle of more than 120° and an H-A-X angle of more than 90° are indicative of an A-X atom configuration. Three types of hydrophobic interactions are indicated by the color purple: pi-pi stacking, which involves two aromatic groups stacked face-to-face with a distance between centroids of less than 4.4 Å and an angle between planes of less than 30°, or face-to-edge with a distance between centroids of less than 5.5 Å and an angle between planes of greater than 60°; pi-cation, which involves aromatic and charged group centroids within 4.5 Å; and general, which involves a hydrophobic side chain within 3.6 Å of an aromatic or aliphatic carbon of the ligand.

**Figure 6:**
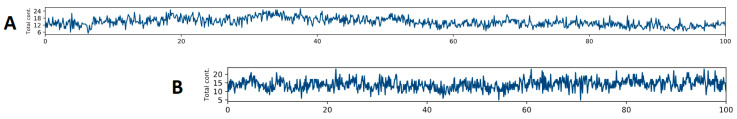
The total average number of interactions over the simulation’s duration for MOR (**A**) and D2R (**B**). The protein–ligand contacts are calculated based on the whole 100 ns trajectories.

Figure 5 and Figure 6 show protein–ligand interaction graphs. It should be mentioned that the interactions found in the MD simulations are not the same as those found in molecular docking. This is because molecular docking did not include water molecules in the system that was exposed to MD simulations. Additionally, in the instance of the complex with D2R, the simulation allowed for the study of the ligand’s conformational changes as well as the development of intermolecular interactions between the various functional groups of LENART01. Also, in the case of the complex with D2R, the ratio of hydrophobic interactions was significantly larger than that in MOR.

To analyze the behavior of the ligand in the form of a complex, Figure S11 was created. The RMSD for the ligand was more significant in the complex with D2R. In both studied complexes, the plateau of RMS was reached after ca. 60 ns, which agrees with the RMSD for protein.

The ligand’s radius of gyration (RoG) was larger in the complex with MOR. An increase in RoG values implies a decrease in peptide structure compactness, thereby suggesting increased flexibility and less stability.

The number of intramolecular hydrogen bonds in the ligand was higher in the complex with D2R.

The polar surface area (PSA), solvent-accessible surface area (SASA) and molecular surface area (MolSA) of the ligand were observed to be larger in the simulation of the complex with MOR. This was a direct consequence of the larger RoG of the ligand in this complex, and indicates less tight packing than that of the same ligand in the complex with D2R.

## 3. Materials and Methods

### 3.1. Drugs and Reagents

LENART01 was synthesized as described elsewhere [8]. In the case of receptor binding assays, the GTP analog GTPγS and GDP were purchased from Sigma-Aldrich (Budapest, Hungary). The highly selective MOR agonist Tyr-D-Ala-Gly-(NMe)Phe-Gly-ol (DAMGO) and the selective MOR agonist dermorphin were both obtained from Bachem Holding AG (Bubendorf, Switzerland). The non-selective opioid receptor antagonist naloxone was kindly provided by the company Endo Laboratories DuPont de Nemours (Wilmington, DE, USA). SCH-23390, a selective dopamine receptor DRD1 antagonist, and the selective DRD2 antagonist, spiperone, were obtained from Tocris Bioscience (Bristol, UK). The radiolabeled [^35^S]GTPγS with specific 1000 Ci/mmol activity was purchased from Hartmann Analytic (Braunschweig, Germany). Similarly, [^3^H]DAMGO (38.8 Ci/mmol) was radiolabeled in the Isotope Laboratory of BRC (Szeged, Hungary), while [^3^H]SCH−23390 (83.2 Ci/mmol) and [^3^H]Spiperone (80.2 Ci/mmol) were obtained commercially from PerkinElmer (Boston, MA, USA).

### 3.2. NMR

#### 3.2.1. Sample Preparation

The NMR sample was prepared by dissolving the 5.6 mg peptide in 550 μL DMSO-d_6_ solvent. Considering the molecular mass of the studied peptide (1276 Da), the concentration in the NMR sample can be estimated as 8 mM.

#### 3.2.2. NMR Measurements

The NMR experiments were performed on a Varian Inova 500 spectrometer equipped with three channels together with a *z* gradient unit. NMR data were acquired by utilizing a ^1^H/^13^C/^15^N cryogenic probehead with inverse detection. All spectra were calibrated to the DMSO-d_6_ signals observed at 2.50 ppm and 39.5 ppm in the ^1^H and ^13^C dimensions, respectively. To perform a structural analysis of the peptide in solution, the two-dimensional homonuclear experiments ^1^H-^1^H TOCSY (with mixing time 80 ms) and ^1^H-^1^H ROESY (with mixing time 400 ms) were conducted. These data were supplemented by heteronuclear ^1^H-^13^C HSQC and ^1^H-^15^N HSQC experiments collected on the natural abundance of the ^13^C and ^15^N resonances. All collected NMR spectra were processed with NMRPipe [20] and analyzed using Sparky [21] software (https://www.cgl.ucsf.edu/home/sparky/ (accessed on 7 September 2020)).

#### 3.2.3. Structure Calculations

Three-dimensional structure calculations were performed with CYANA (version 3.98.13) software [22]. The ^1^H-^1^H distance constraints were yielded from the analysis of the ROESY spectrum. The additional restraints to the backbone φ and ψ torsion angles were deduced from ^1^H, ^13^C and ^15^N chemical shifts with TALOSn software (http://spin.niddk.nih.gov/bax/software/TALOS-N/ (accessed on 19 March 2014)) [12]. The conformation of the Tyr5–Pro6 was predicted as *trans* based on chemical shifts by PROMEGA [23] and later confirmed with observed cross-peaks between ^1^H^α^ Tyr5 and ^1^H^δ^ Pro6.

### 3.3. Receptor Binding Assay Ex Vivo

#### 3.3.1. Animals

Both male and female Wistar rats were used for membrane preparations (brains and spinal cords). Animals were housed at 22 ± 0.5 °C with a 12:12 h light–dark cycle. Food and water were available *ad libitum*. All animal manipulations were carried out according to the European Communities Council Directives (86/606/ECC) and the Hungarian Act for the Protection of Animals in Research (XXVIII.tv. 32.§).

#### 3.3.2. Competitive Binding Experiments

Competitive analysis of the chimera activity was performed in the presence of naloxone (in case of MOR displacement studies) or SCH-23390 (for D1R) and spiperone (for D2R). For MOR displacement, aliquots of rat brain and spinal cord membrane homogenates were thawed and suspended in 50 mM Tris-HCl buffer (pH 7.4). In the case of D1R displacement, the Tris-HCl buffer (pH 7.4) contained additional ingredients, i.e., 120 mM NaCl, 5 mM KCl, 1 mM MgCl_2_, 2 mM CaCl_2_ and 1 µM mianserin, whereas in the D2R displacement, the Tris-HCl buffer (pH 7.4) contained 5 mM KCl, 2 mM MgCl_2_, 2 mM CaCl_2_ and 1 µM ketanserin. In each case, membranes were incubated in the presence of the unlabeled ligands at increasing concentrations (10^−10^–10^−5^ M) with [^3^H]DAMGO, [^3^H]SCH-23390 and [^3^H]Spiperone; 10 µM unlabeled naloxone or dopaminergic receptor antagonists defined non-specific binding in the competition assays. The reaction was terminated by rapid filtration under vacuum, followed by washes with ice-cold Tris-HCl. The radioactivity of the dried filters was detected using an UltimaGold^TM^ MV aqueous scintillation cocktail with a Packard Tricarb 2300TR liquid scintillation counter. The competitive binding assays were performed in duplicate and repeated three times.

#### 3.3.3. Functional [^35^S]GTPγS Binding Experiments

The functional [^35^S]GTPγS binding experiments were performed as previously described [11]. In brief, the brain and spinal cord homogenates were incubated at 30 °C for 60 min in Tris-EGTA buffer (pH 7.4) containing [^35^S]GTPγS (0.05 nM) and in the presence of varying concentrations of the ligand. Non-specific binding was determined in the presence of non-radiolabeled 10 μM GTPγS. The reaction was stopped by rapid filtration under vacuum (Brandel M24R Cell Harvester), and the samples were washed with cold Tris-HCl buffer. The radioactivity of the dried filters was determined by scintillation counting.

### 3.4. Molecular Modeling

Molecular docking, MM-GBSA calculations and molecular dynamics (MD) simulations were performed using different modules of Schrӧdinger Maestro version 12.8. (Schrӧdinger, LLC., New York, NY, USA, 2023).

#### 3.4.1. Structures Preparation

Three-dimensional crystal structures of two proteins—a human-active μ-opioid receptor (MOR), bound to the agonist BU72 at 2.07 Å resolution (PDB ID: 5C1M), and dopamine receptor 2 (D2R), bound to the agonist (bromocriptine) in complex with Gi protein at 2.80 Å resolution (PDB ID: 7JVR)—were retrieved from the RCBS PDB database [24]. It should be noted that only recently (2021) has the structure of the D2R with its agonist been obtained and deposited. The structure of D2R has been crystallized with its Gi subunit (residues starting from 589). Therefore, the number of residues in 7JVR is significantly larger than in 5C1M. Those structures were cleaned up with the Protein Preparation Wizard to remove extraneous cofactors, metal ions and water molecules before docking. In addition, disulfide bonds were formed, bond orders were appropriately assigned, ionization states were adjusted, multimeric complexes were simplified and misoriented groups were corrected in orientation. The protein structures were modified by adding hydrogen atoms, and standard protonation states at pH 7.0 were applied. Geometrically stable structures were then produced by optimizing and minimizing the preprocessed structures. Following preparation, the protein structures were used to continue modeling.

#### 3.4.2. Protein–Peptide Docking

##### Active Site Identification and Grid Generation

The grid center, in every instance, was the mass center of the co-crystallized ligand (either BU72 or bromocriptine). A cubic search box was created, and the grid size was adjusted to fit peptides of 11 residues, such as LENART01 (YdAFGYPSGHFM), completely. Using the OPLS 2005 force field, receptor grids were created using the default values for the charge cutoff (0.25) and van der Waals scaling factor (1.00).

##### Ligand Preparation

LigPrep from the Schrödinger Suite was used to prepare the investigated peptide LENART01, YdAFGYPSGHFM, for docking: protonation states were created at pH 7.4 ± 2.0 while maintaining the chiralities. The force field utilized for the geometry optimization was OPLS 2005. The default values for LigPrep’s other options were maintained.

##### Glide SP–Peptide Docking

Flexible protein–peptide docking was performed using the grid-based ligand docking with energetics (Glide) module and SP–peptide precision scheme. Glide has a special peptide docking mode (SP–peptide) designed to handle the much greater flexibility of peptides relative to the usual ligands. This mode has enhanced sampling and other settings, enabling it to capture a wider range of poses. The first Glide docking stage’s default 20 postures were kept for the docking. The formula for calculating the glide score (GScore) was GScore = 0.065 × vdW + 0.130 × Coul + Lipo + Hbond + Metal + BuryP + RotB + Site. Here, vdW stands for van der Waals energy, Coul for Coulomb energy, Lipo for lipophilic term, Hbond for hydrogen bonding, Metal for metal-binding term, BuryP for Buried Polar groups’ penalty, RotB for penalty for frozen rotatable bonding and Site for active-site polar interactions. GlideScore, the non-bonded interaction energy, and, in the case of flexible docking, the excess internal energy of the formed ligand conformation were combined to create an Emodel.

#### 3.4.3. Binding Free Energy Calculation by MM/GBSA Rescoring

The calculation of binding free energy (ΔG_bind_) values was exploited to estimate in silico ligand binding affinities. The molecular mechanics/generalized Born surface area (MM/GBSA) rescoring method was used to calculate binding free energies accurately [25]. For this purpose, the Prime MM/GBSA module was used.

MM/GBSA rescoring was carried out for both the initial ligand-docked poses with the highest scoring functions and snapshots from 100 ns MD simulation. Each MD simulation trajectory yielded 1000 images, from which average ΔGbind values were determined.

With the use of the VSGB solvent model and the OPLS 2005 force field, the free energy changes that occurred during protein–ligand interactions were determined.

The values of binding free energy were computed using the subsequent formula:

MM/GBSA ΔG_bind_ = G_complex(optimized)_ − (G_protein(optimized)_ + G_peptide(optimized)_)

The following method was used to estimate the free energy of each state—that is, the complex, protein and peptide—by taking into account solvation energies, entropic terms and molecular mechanics energies:

G = G_int_ + G_Coulomb_ +G_vdW_ + G_GB_ + G_lipo_ − TS

where T is an absolute temperature; S is an entropy value; and G_int_, G_Coulomb_ and G_vdW_ are standard MM energy terms for bond (covalent, angle and dihedral), Coulomb (electrostatic) and van der Waals interactions. G_GB_ and Gl_ipo_ are the polar and non-polar (lipophilic) contributions to the solvation free energies. While the non-polar contribution (G_lipo_) was estimated using the solvent-accessible surface area (SASA), the polar contribution (G_GB_) was computed using the generalized Born model.

#### 3.4.4. Molecular Dynamics (MD) Simulations

An input for the MD simulations was the ligand–receptor complexes that yielded the best docking scores. The module for the Desmond System Builder was utilized for that purpose. The orthorhombic boxes held the peptide–protein complexes embedded in a DPPC membrane that had previously been adjusted. Using a buffer distance of 10 Å and the TIP3P water model [26], the systems were solvated with water. In each instance, the system was rendered neutral by the addition of the proper quantity of Cl-ions. Before running MD simulations, the systems were implemented via Desmond’s default protocol’s steepest descent minimization. The relaxation protocol was divided into eight stages: simulation with heating from 0 K to 300 K, simulation with restraints on solute heavy atoms, simulation under NPT equilibration with H_2_O barrier and gradual restraining, simulation under NPT equilibration of solvent and lipids, simulation under the NPT ensemble with protein heavy atoms’ restraint reduced from 10.0 to 2.0 kcal/mol, simulation under NPT equilibration with C_α_ atoms restrained at 2 kcal/mol and simulation under 1.5 ns under the NPT ensemble without any restraints. Following relaxation, each system had an unrestricted simulation run lasting 100 ns. Under the NPT ensemble, the simulations were run using an isotropic Martyna–Tobias–Klein barostat set to maintain a pressure of one atm and a Nose–Hoover thermostat set to maintain a constant temperature of 300 K. Using the short-range approach, the short-range Coulombic interactions were examined using a cut-off value of 9.0 Å. With a time-step of 2.0 fs, a time-reversible reference system propagator algorithm (RESPA) integrator was employed. For analysis, the trajectories were stored at 100 ps intervals. A simulation interaction diagram from the Schrödinger Suite was used to assess protein–ligand interactions, RMSD and RMSF following the completion of the simulations. Interaction fingerprints (IFPs) were created by encoding interactions that occurred in every frame of the simulations that were run.

## 4. Conclusions

Designed peptides and/or proteins that can fold into secondary structures continue to be valuable tools in studying protein structure–function relationships. Molecular modeling methods revealed the interactions between the studied receptors and LENART01 in the analyzed complexes. The calculated values of both Glide docking scores and MM/GBSA binding free energy values confirm the energetic and thermodynamic stability of the studied systems. Furthermore, these studies confirmed the in vitro binding studies of the chimera, providing additional useful information on its potent behavior concerning the interaction with the target molecule of interest.

## Figures and Tables

**Table 1:** Functional binding data of LENART01 at opioid and dopamine receptors.

	Ligand		E_max_ ± S.E.M. (%)		
			Opioid System	Dopaminergic System	
			Ligand + Cyprodime	Ligand + SCH-39166	Ligand + Risperidone
Brain membrane	dermorphin	140.5 ± 2.9	100.0 ± 1.1 ***	n.d.^a^	n.d.^a^
	chimera	140.6 ± 1.0	100.4 ± 1.4 ***	140.8 ± 0.8 ^ns^	101.1 ± 0.7 ***
	ranatensin	124.1 ± 1.9	n.d.^a^	119.1 ± 0.8 ***	101.5 ± 1.7 ***
Spinal cord membrane	dermorphin	140.0 ± 1.8	100.4 ± 0.9 ***	n.d.^a^	n.d.^a^
	chimera	136.3 ± 2.2	100.8 ± 1.1 ***	136.1 ± 1.7 ^ns^	101.6 ± 1.2 ***
	ranatensin	119.8 ± 1.3	n.d.^a^	117.4 ± 1.1 ^ns^	100.0 ± 0.7 ***

^a^: not determined. Experimental data were processed by GraphPad Prism 5.0 using bar graphs. ^ns^: not significant; ***: *p* < 0.001 indicates a significant difference between ligands and ligand(s) + receptor antagonist; the statistical significances were determined based on unpaired *t*-tests.

**Table 2:** Docking scores, MM-GBSA ΔG of binding and type of interactions in the complexes formed between the chimera with a sequence of YdAFGYPSGHFM and D2R (PDB: 7JVR) or MOR (PDB: 5C1M).

	Glide G-Score (kcal/mol)	Glide Emodel (kcal/mol)	MM/GBSA ΔG Binding (kcal/mol)	Residues Forming Hydrogen Bonds	Residues Forming π Interactions
MOR (5C1M)	−10.343	−147.199	−23.53	LYS209, ARG211, PHE221, SER222, THR225	HIS223
D2R (7JVR)	−13.800	−205.959	−48.75	CYS182, ASP400, CYS401	HIS393

**Table 3:** Particular contributions to the total MM/GBSA energy from different components in the complexes formed between the chimera with a sequence of YdAFGYPSGHFM and D2R (PDB: 7JVR) or MOR (PDB: 5C1M). All of the values are in kcal mol^−1^.

	MM/GBSA ΔG Binding	CE	CB	HB	LE	PP	GB	SA
MOR (5C1M)	−23.53	8.72	−11.12	−2.86	−5.81	0.75	36.09	−49.30
D2R (7JVR)	−48.75	−97.26	−2.39	−1.24	−34.51	−0.26	164.69	−77.78

CE: Coulomb energy; CB: covalent binding energy; HB: hydrogen bonding correction; LE: lipophilic energy; PP: pi-pi packing correction; GB: generalized Born electrostatic solvation energy; SA: surface area electrostatic solvation energy.

## Data Availability

The data presented in this study are available on request from the corresponding author.

## Supplementary Materials

The following supporting information can be downloaded at: https://www.mdpi.com/article/10.3390/molecules29010272/s1, Table S1. ^1^H, ^13^C and ^15^N chemical shifts assigned for resonances in DMSO-d_6_, Figure S1. ^1^H-^13^C HSQC experiment performed with parameters tuned to aliphatic resonances. The resonances ^13^CH_3_ (methyl) and ^13^CH groups are highlighted in red, the resonances from ^13^CH_2_ groups are shown in green, Figure S2. Aromatic fragment of ^1^H-^13^C HSQC spectrum. Assignments of the ^1^H and ^13^C resonances for two tyrosines (Tyr1, Tyr5), two phenylalanines (Phe3, Phe10) and histidine (His9) are shown, Figure S3. Fragment of the 2D homonuclear ^1^H-^1^H ROESY spectrum recorded with mixing time 400 ms at 298 K, Figure S4. Results of analysis of φ and ψ backbone torsion angles on base ^1^H, ^13^C and ^15^N chemical shifts for the Phe3, Gly4, Ser7 and His9 by the program TALOSn. The α-helical conformation was detected for all presented residues, Figure S5. Result of analysis of the backbone conformation (Ramachandran plot) for the chimera peptide performed by the program Procheck, Figure S6. Various representations of LENART01 3D structure in DMSO-d_6_ solution. (A) An ensemble of 20 structures of the LENART01 is shown, including the conformation of the side chains for all residues. The positions of protons are also visible. (B) The same ensemble is presented in different views. For clarification reasons, the protons are hidden. In side chains, only bonds between heavy atoms are highlighted. In both views, the alpha-helical conformation in the fragment Tyr5–Met11 is detected, Figure S7. RMSD plot obtained for Cα-atoms of MOR in the complex formed between YdAFGYPSGHFM and MOR during 100 ns MD simulation, Figure S8. RMSD plot obtained for Cα-atoms of D2R in the complex formed between YdAFGYPSGHFM and D2R during 100 ns MD simulation, Figure S9. RMSF plot obtained for Cα-atoms of (A) MOR in the complex formed between YdAFGYPSGHFM and MOR and (B) D2R in the complex formed between the chimera and D2R, respectively, during 100 ns MD simulation. The secondary structure elements: strand (blue) and helix (pink), Figure S10. Secondary structure content (SSE, %) of the MOR (A) and the D2R (B) as a function of residue number, and for each trajectory frame over the course of the simulation of the MOR (C) and the D2R (D). The secondary structure elements: strand (blue) and helix (red), Figure S11. Plots and bar charts of six ligand properties during the simulation (from the bottom): polar surface area (PSA), solvent-accessible surface area (SASA), molecular surface area (MolSA), intramolecular hydrogen bonds (intraHB), radius of gyration (rGyr) and ligand RMSD with respect to the initial conformation. For each property, there is a chart that shows the value of the property as a function of time; to the right, there is a bar chart that shows the proportion of time spent in each of 10 value ranges, divided equally over the range of property values. The subsequent sets of plots and bars are presented for the MOR (A) and the D2R (B).

## References

[1] Nepali K., Sharma S., Sharma M., Bedi P., Dhar K. (2014). Rational approaches, design strategies, structure activity relationship and mechanistic insights for anticancer hybrids. Eur. J. Med. Chem..

[2] Kleczkowska P., Hermans E., Kosson P., Kowalczyk A., Lesniak A., Pawlik K., Bojnik E., Benyhe S., Nowicka B., Bujalska-Zadrozny M. (2016). Antinociceptive effect induced by a combination of opioid and neurotensin moieties vs. their hybrid peptide [Ile(9)]PK20 in an acute pain treatment in rodents. Brain Res..

[3] Lipinski C.A., Lombardo F., Dominy B.W., Feeney P.J. (1997). Experimental and computational approaches to estimate solubility and permeability in drug discovery and development settings. Adv. Drug Deliv. Rev..

[4] Nygaard R., Zou Y., Dror R.O., Mildorf T.J., Arlow D.H., Manglik A., Pan A.C., Liu C.W., Fung J.J., Bokoch M.P. (2013). The dynamic process of beta(2)-adrenergic receptor activation. Cell.

[5] Chen K.Y., Zhou F., Fryszczyn B.G., Barth P. (2012). Naturally evolved G protein-coupled receptors adopt metastable conformations. Proc. Natl. Acad. Sci. USA.

[6] Chen Y.C. (2015). Beware of docking!. Trends Pharmacol. Sci..

[7] Mollica A., Pelliccia S., Famiglini V., Stefanucci A., Macedonio G., Chiavaroli A., Orlando G., Brunetti L., Ferrante C., Pieretti S. (2017). Exploring the first Rimonabant analog-opioid peptide hybrid compound, as bivalent ligand for CB1 and opioid receptors. J. Enzym. Inhib. Med. Chem..

[8] Serafin P., Kowalczyk P., Mollica A., Stefanucci A., Laskowska A.K., Zawadzka M., Kramkowski K., Kleczkowska P. (2023). Evaluation of antimicrobial activities against various E. coli strains of a novel hybrid peptide—LENART01. Molecules.

[9] Laskowska A.K., Szudzik M., Ścieżyńska A., Komorowski M., Szucs E., Gombos D., Bączek B., Lipka-Miciuk J., Benyhe S., Kleczkowska P. (2022). The role of a natural amphibian skin-based peptide, ranatensin, in pancreatic cancer expressing dopamine D2 receptors. Cancers.

[10] Zhu X.Z., Ji X.Q., Wu S.X., Zou G. (1991). Sulpiride attenuates ranatensin-M-induced antinociception. Zhongguo Yao Li Xue Bao.

[11] Bird M.F., Cerlesi M.C., Brown M., Malfacini D., Vezzi V., Molinari P., Micheli L., Mannelli L.D.C., Ghelardini C., Guerrini R. (2016). Characterisation of the novel mixed Mu-NOP peptide ligand dermorphin-N/OFQ (DeNo). PLoS ONE.

[12] Shen Y., Bax A. (2013). Protein backbone and sidechain torsion angles predicted from NMR chemical shifts using artificial neural networks. J. Biomol. NMR.

[13] Piela L., Némethy G., Scheraga H.A. (1987). Proline-induced constraints in alpha-helices. Biopolymers.

[14] Dallavalle S., Dobričić V., Lazzarato L., Gazzano E., Machuqueiro M., Pajeva I., Tsakovska I., Zidar N., Fruttero R. (2020). Improvement of conventional anti-cancer drugs as new tools against multidrug resistant tumors. Drug Resist. Updat..

[15] Singh A.K., Kumar A., Singh H., Sonawane P., Paliwal H., Thareja S., Pathak P., Grishina M., Jaremko M., Emwas A.H. (2022). Concept of hybrid drugs and recent advancements in anticancer hybrids. Pharmaceuticals.

[16] Starnowska-Sokół J., Przewłocka B. (2020). Multifunctional opioid-derived hybrids in neuropathic pain: Preclinical evidence, ideas and challenges. Molecules.

[17] Raschka S., Wolf A., Bemister-Buffington J., Kuhn L. (2018). Protein-Ligand interfaces are polarized: Discovery of a strong trend for intermolecular hydrogen bonds to favor donors on the protein side with implications for predicting and designing ligand complexes. J. Comput. Aided Mol. Des..

[18] Martínez L. (2015). Automatic identification of mobile and rigid substructures in molecular dynamics simulations and fractional structural fluctuation analysis. PLoS ONE.

[19] Liu K., Watanabe E., Kokubo H. (2017). Exploring the stability of ligand binding modes to proteins by molecular dynamics simulations. J. Comput. Aided Mol. Des..

[20] Delaglio F., Grzesiek S., Vuister G., Zhu G., Pfeifer J., Bax A. (1995). NMRPipe: A multidimensional spectral processing system based on UNIX pipes. J. Biomol. NMR.

[21] Lee W., Tonelli M., Markley J.L. (2015). NMRFAM-SPARKY: Enhanced software for biomolecular NMR spectroscopy. Bioinformatics.

[22] Guntert P. (2004). Automated NMR protein structure calculation with CYANA. Methods Mol. Biol..

[23] Shen Y., Bax A. (2010). Prediction of Xaa-Pro peptide bond conformation from sequence and chemical shifts. J. Biomol. NMR.

[24] Berman H.M., Westbrook J., Feng Z., Gilliland G., Bhat T.N., Weissig H., Shindyalov I.N., Bourne P.E. (2000). The Protein Data Bank. Nucleic Acids Res..

[25] Genheden S., Ryde U. (2015). The MM/GBSA methods to estimate ligand-binding affinities. Expert. Opin. Drug Discov..

[26] Jorgensen W.L., Chandrasekhar J., Madura J.D., Impey R.W., Klein M.L. (1983). Comparison of simple potential functions for simulating liquid water. J. Chem. Phys..

