# Supplementary material for: Opioid/Dopamine Receptor Binding Studies, NMR and Molecular Dynamics Simulation of LENART01 Chimera, an Opioid-Bombesin-like Peptide
Source: Molecules. 2024 Jan 4;29(1):272. doi: 10.3390/molecules29010272 (PMC10780910; doi:10.3390/molecules29010272)
Supplement: Supplementary file 1 [file molecules-29-00272-s001.zip › molecules-2796170-supplementary.pdf]

Supplementary Materials

Table S1. ^{1}H, ^{13}C, and ^{15}N chemical shifts assigned for resonances in DMSO-d_6.

Residue	$^{15}N/^{1}HN$	$^{13}C^{\alpha}/^{1}H^{\alpha}$	$^{13}C^{\beta}/^{1}H^{\beta}$	$^{13}C/^{1}H$
Tyr1		61.2/3.948	44.3/2.829	$^{13}C^{\delta}/^{1}H^{\delta}$ 133.7/6.980,<br>$^{13}C^{\varepsilon}/^{1}H^{\varepsilon}$ 118.5/6.671
D-Ala2	125.8/8.382	55.7/4.340	26.6/0.706	
Phe3	117.6/8.385	61.4/4.576	46.1/2.999,2.667	$^{13}C^{\delta}/^{1}H^{\delta}$ 131.1/7.237<br>$^{13}C^{\varepsilon}/^{1}H^{\varepsilon}$ 132.5/7.198
Gly4	105.9/8.299	49.3/3.791,3.597		
Tyr5	118.4/8.269	59.2/4.580	44.1/2.866,2.628	$^{13}C^{\delta}/^{1}H^{\delta}$ 133.4/7.058<br>$^{13}C^{\varepsilon}/^{1}H^{\varepsilon}$ 118.2/6.644
Pro6		67.4/4.420	36.7/1.191,1.893	$^{13}C^{\gamma}/^{1}H^{\gamma}$<br>32.4/1.890,1.841<br>$^{13}C^{\delta}/^{1}H^{\delta}$<br>54.8/3.615,3.446
Ser7	112.8/8.002	63.1/4.278	69.5/3.639,3.577	
Gly8	107.1/8.084	50.0/3.755,3.704		
His9	115.5/8.116	60.5/4.582	35.0/3.016,2.888	$^{13}C^{\delta 2}/^{1}H^{\delta 2}$ 120.1/7.258<br>$^{13}C^{\varepsilon 1}/^{1}H^{\varepsilon 1}$ 137.0/8.928
Phe10	118.4/8.160	62.1/4.511	45.1/3.068,2.843	$^{13}C^{\delta}/^{1}H^{\delta}$ 131.4/7.264<br>$^{13}C^{\varepsilon}/^{1}H^{\varepsilon}$ 129.6/7.195
Met11	118.6/8.196	59.8/4.276	39.7/1.945,1.781	$^{13}C^{\gamma}/^{1}H^{\gamma}$ 37.4/2.412<br>$^{13}C^{\varepsilon}/^{1}H^{\varepsilon}$ 22.5/2.032

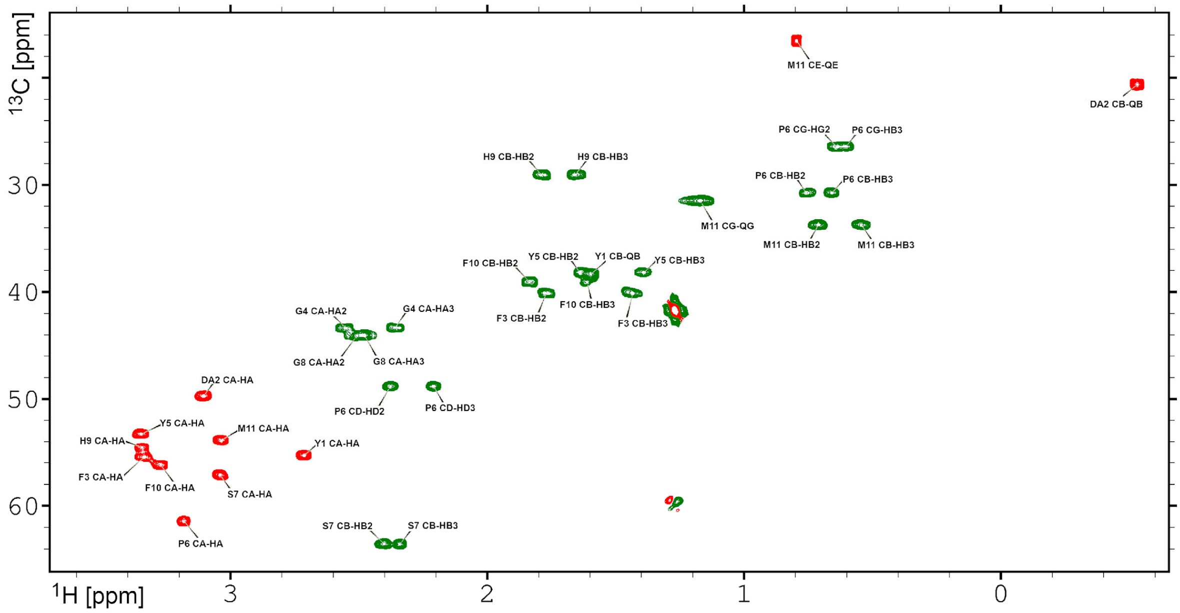

Figure S1. ^{1}H-^{13}C HSQC experiment performed with parameters tuned to aliphatic resonances. The resonances $^{13}CH_3$ (methyl) and ^{13}CH groups are highlighted as red, the resonances from $^{13}CH_2$ groups are shown as green.

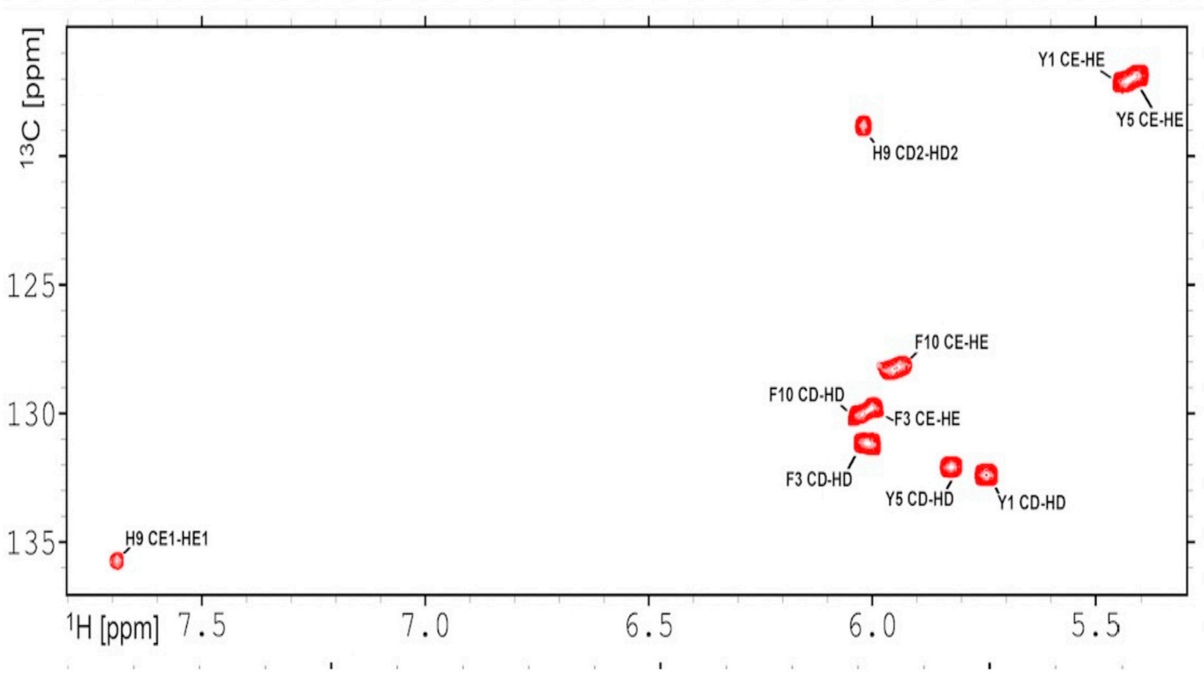

Figure S2. Aromatic fragment of ^{1}H-^{13}C HSQC spectrum. Assignments of the ^{1}H and ^{13}C resonances for two tyrosines (Tyr1, Tyr5), two phenylalanines (Phe3, Phe10), and histidine (His9) are shown.

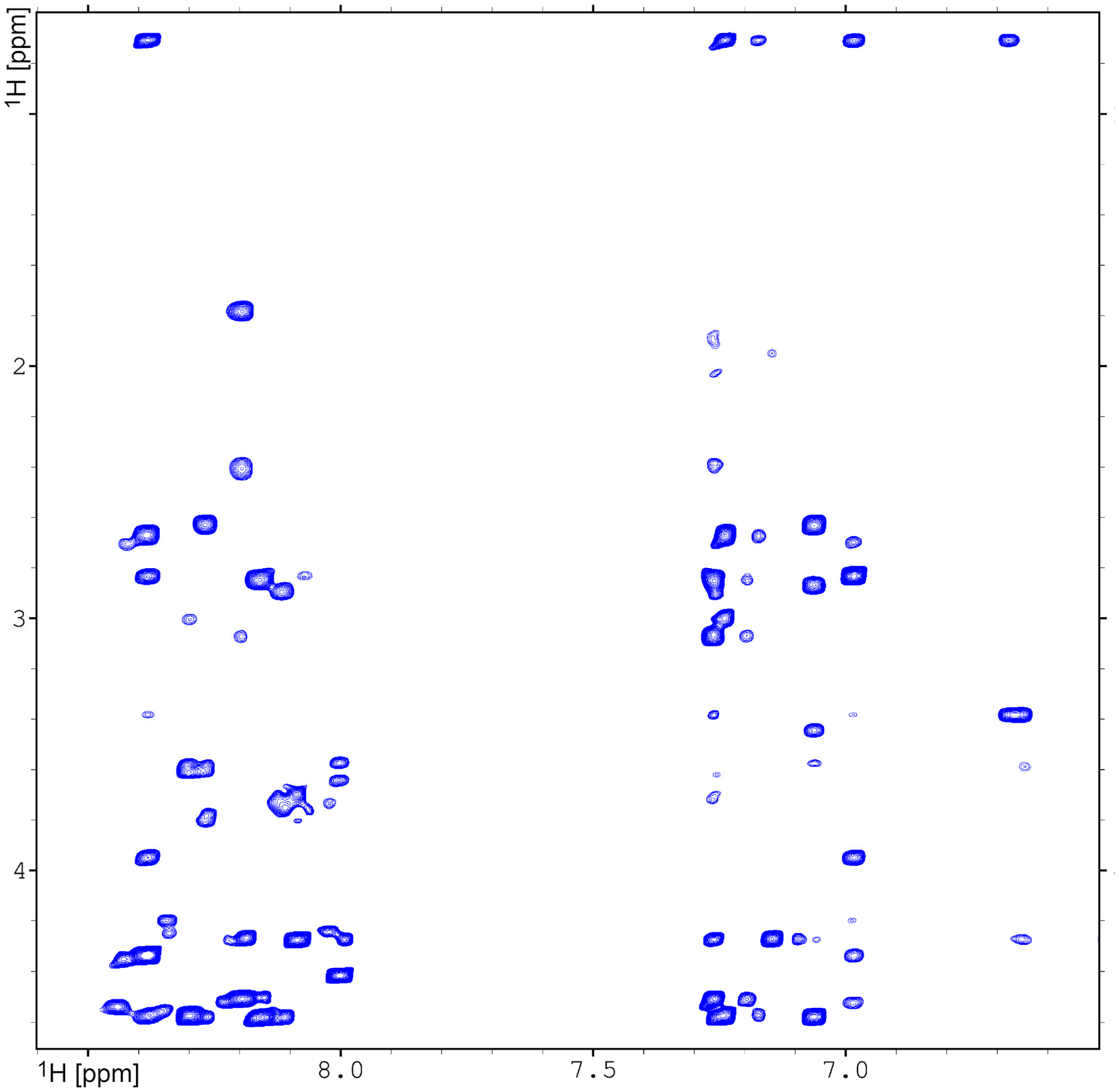

Figure S3. Fragment of the 2D homonuclear ^1H-^1H ROESY spectrum recorded with mixing time 400 ms at 298 K.

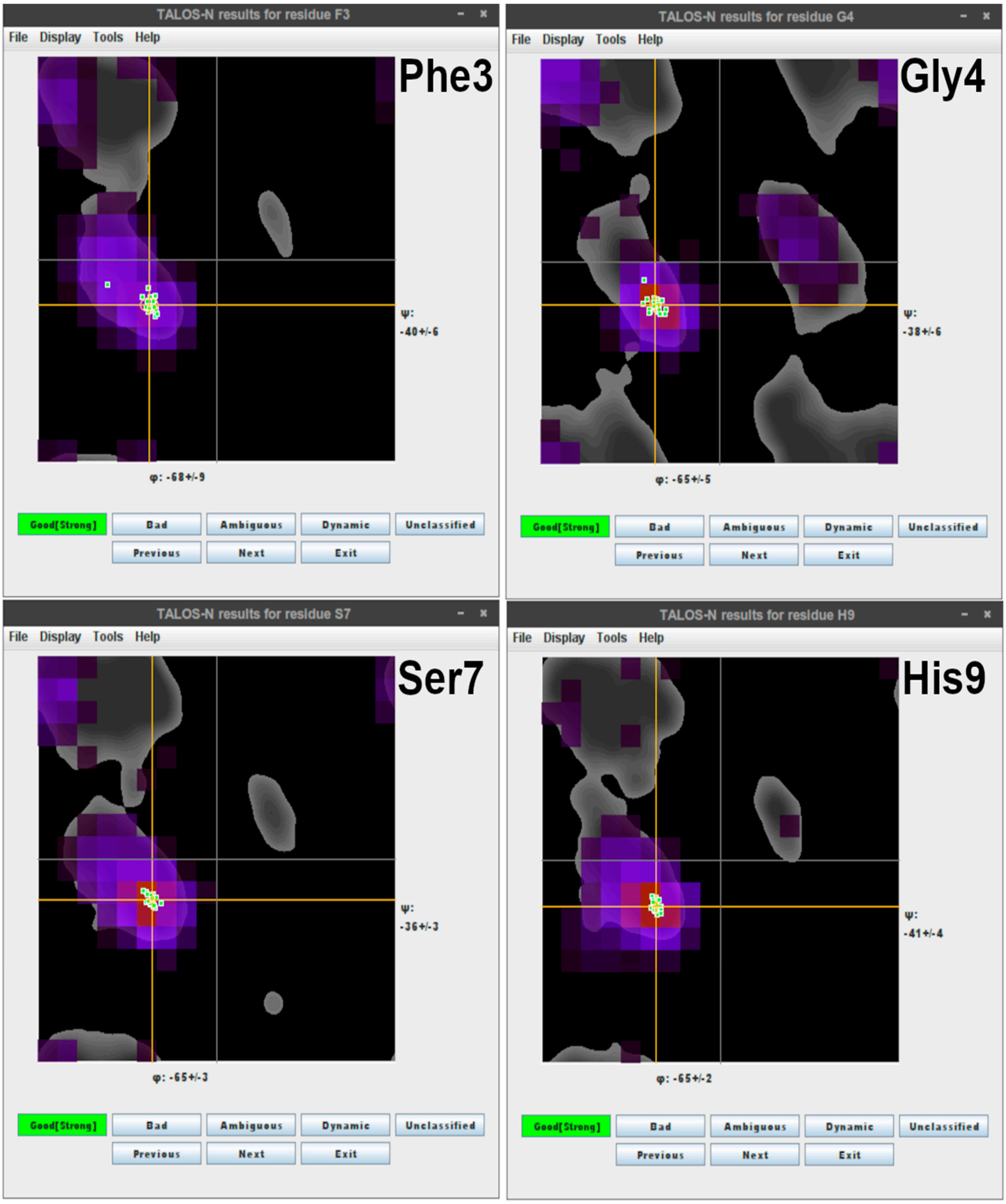

Figure S4. Results of analysis φ and ψ backbone torsion angles on base ^{1}H, ^{13}C, and ^{15}N chemical shifts for the Phe3, Gly4, Ser7, and His9 by the program TALOSn. The α-helical conformation was detected for all presented residues.

PROCHECK-NMR

Ramachandran Plot

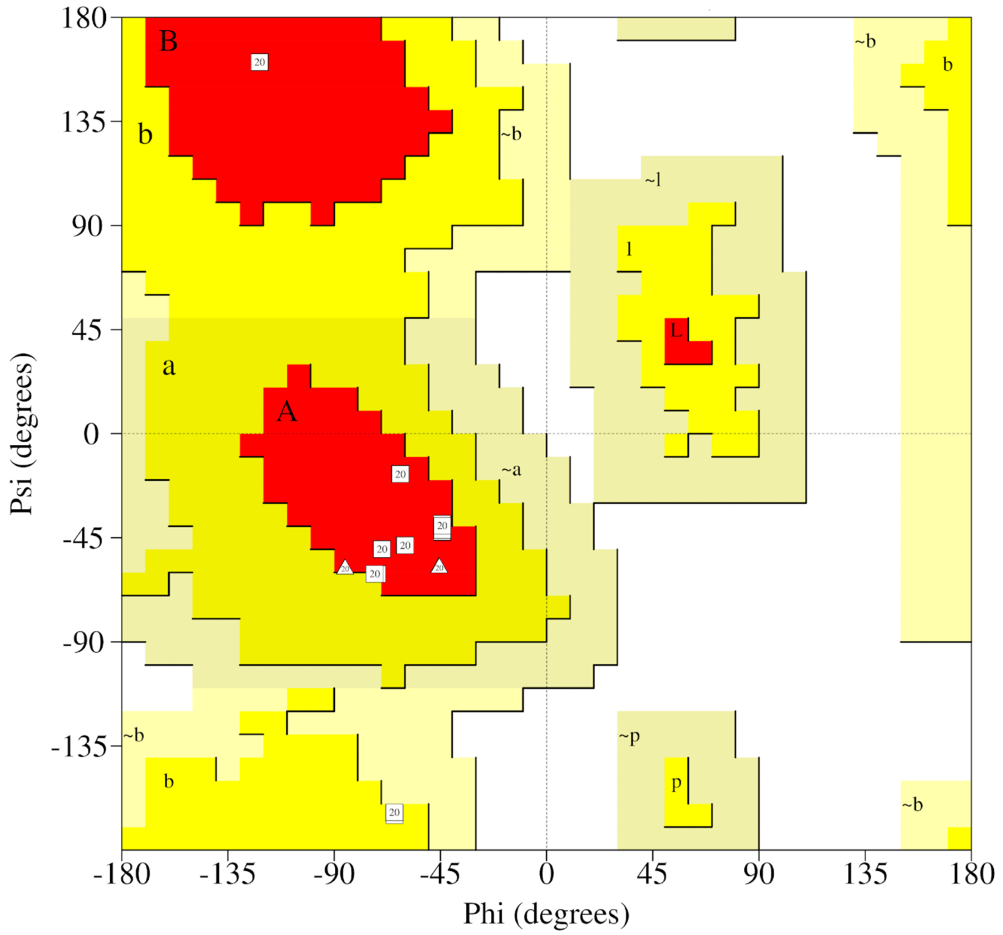

Plot statistics

Residues in most favoured regions [A,B,L]	80	66.7%
Residues in additional allowed regions [a,b,l,p]	40	33.3%
Residues in generously allowed regions [~a,~b,~l,~p]	0	0.0%
Residues in disallowed regions	0	0.0%
	----	------
Number of non-glycine and non-proline residues	120	100.0%
Number of end-residues (excl. Gly and Pro)	40	
Number of glycine residues (shown as triangles)	40	
Number of proline residues	20	

Total number of residues	220	

Figure S5. Result of analysis of the backbone conformation (Ramachandran plot) for chimera peptide performed by the program Procheck.

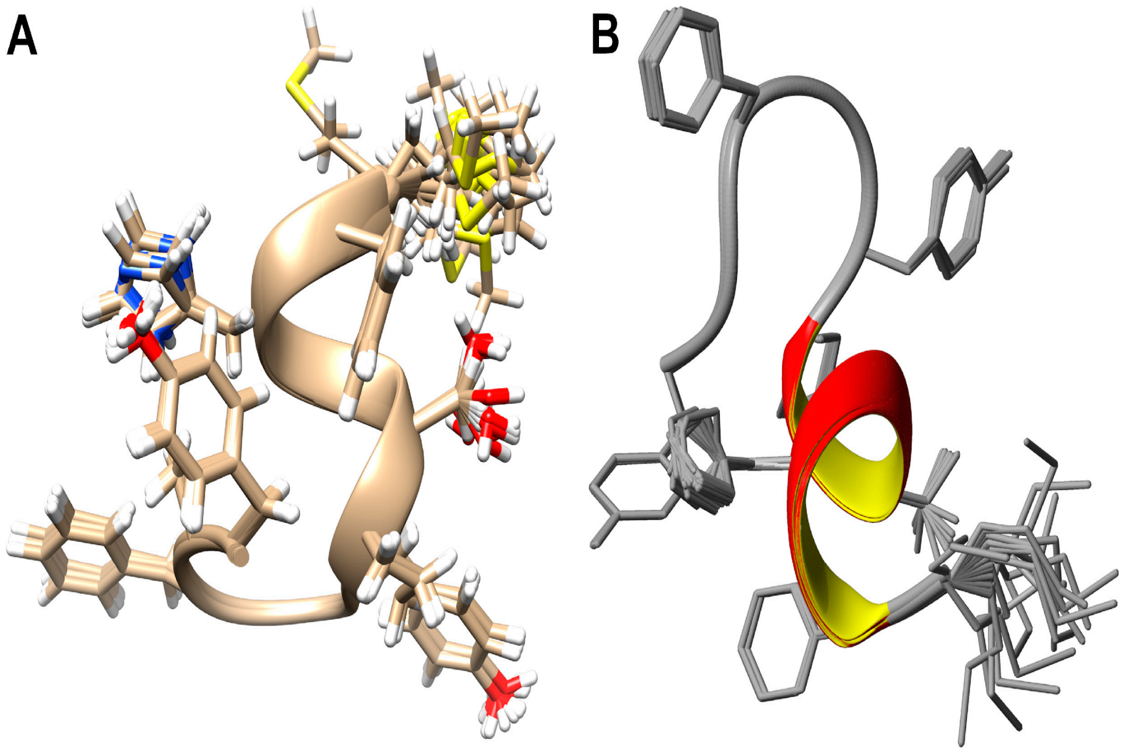

Figure S6. Various representations of LENART01 3D structure in DMSO-d6 solution. (A) An ensemble of 20 structures of the LENART01 is shown, including the conformation of the side chains for all residues. The positions of protons are also visible. (B) The same ensemble is presented in different views. For clarification reasons, the protons are hidden. In side chains, only bonds between heavy atoms are highlighted. In both views, the alpha-helical conformation in the fragment Tyr5 - Met11 is detected.

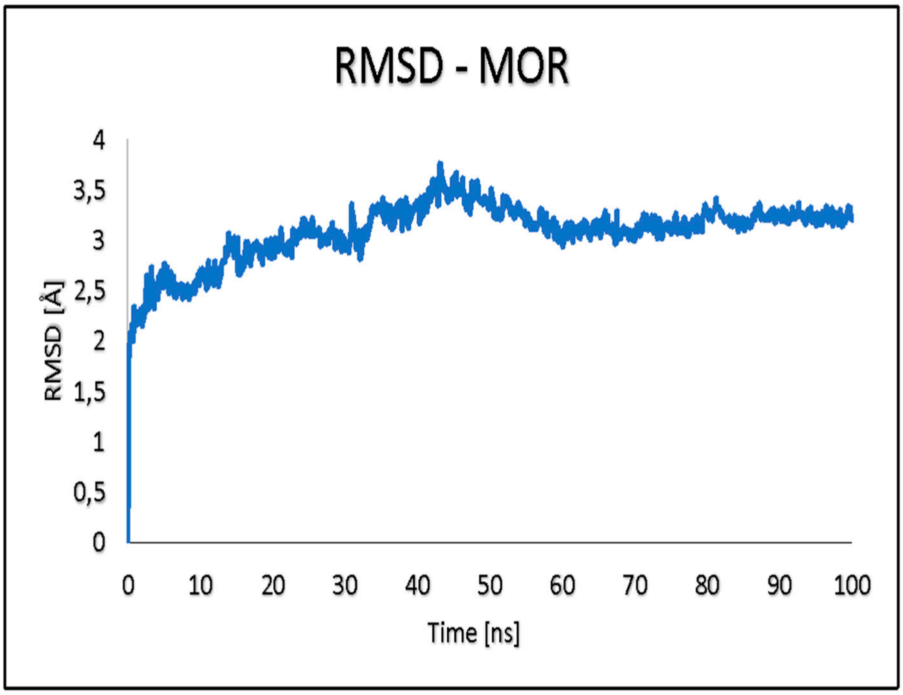

Figure S7. RMSD plot obtained for Cα-atoms of MOR in the complex formed between YdAFGYPSGHFM and MOR during 100 ns MD simulation.

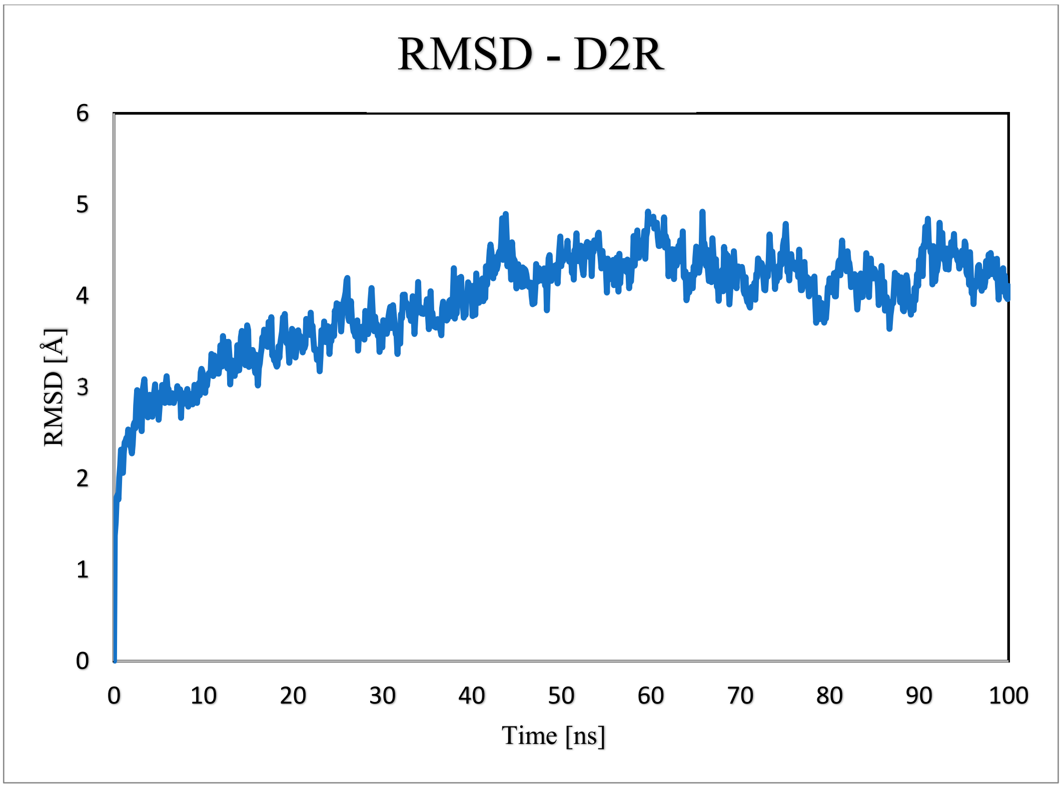

Figure S8. RMSD plot obtained for Cα-atoms of D2R in the complex formed between YdAFGYPSGHFM and D2R during 100 ns MD simulation.

A)

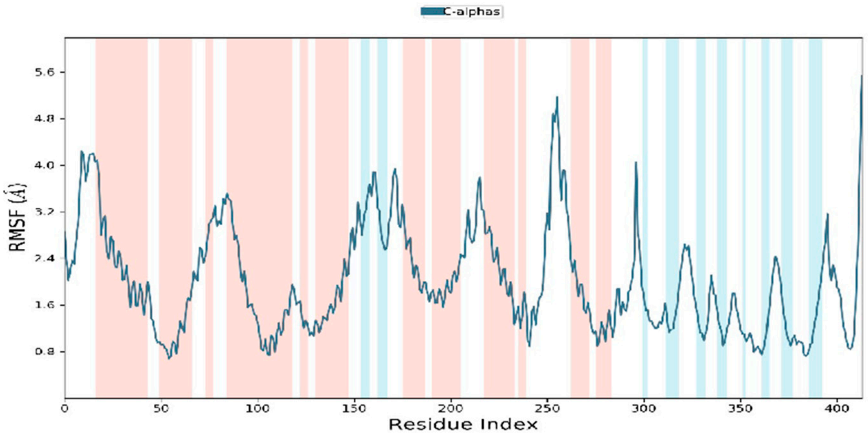

B)

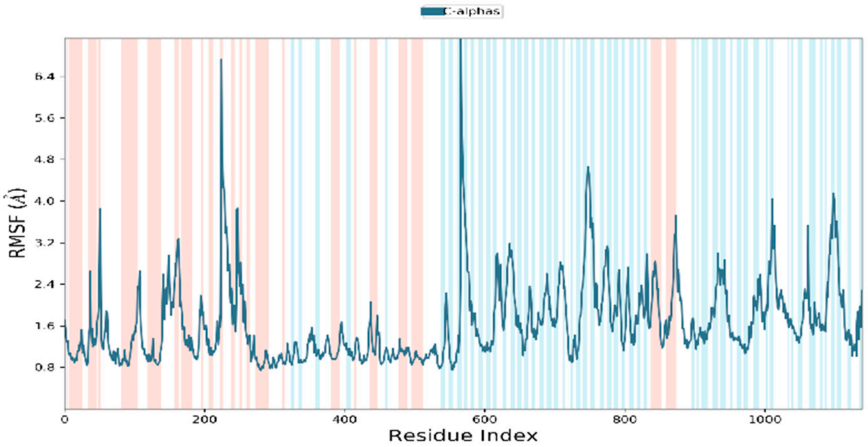

Figure S9. RMSF plot obtained for Cα-atoms of (**A**) MOR in the complex formed between YdAFGYPSGHFM and MOR and (**B**) D2R in the complex formed between the chimera and D2R, respectively, during 100 ns MD simulation. The secondary structure elements: strand (blue) and helix (pink).

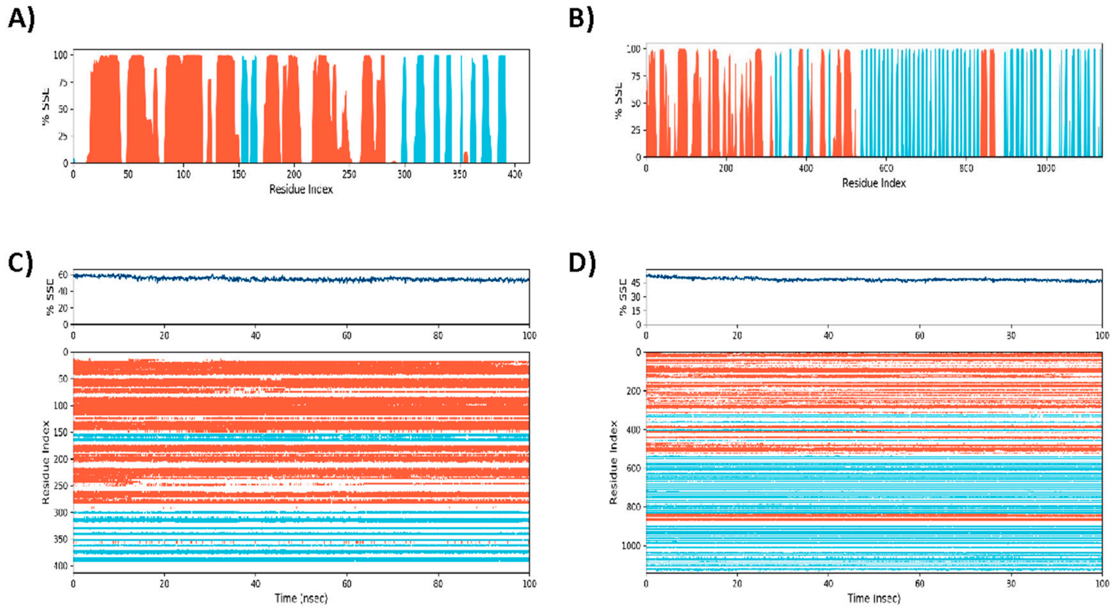

Figure S10. Secondary structure content (SSE, %) of the MOR (A) and the D2R (B) as a function of residue number, and for each trajectory frame over the course of the simulation of the MOR (C) and the D2R (D). The secondary structure elements: strand (blue) and helix (red).

A

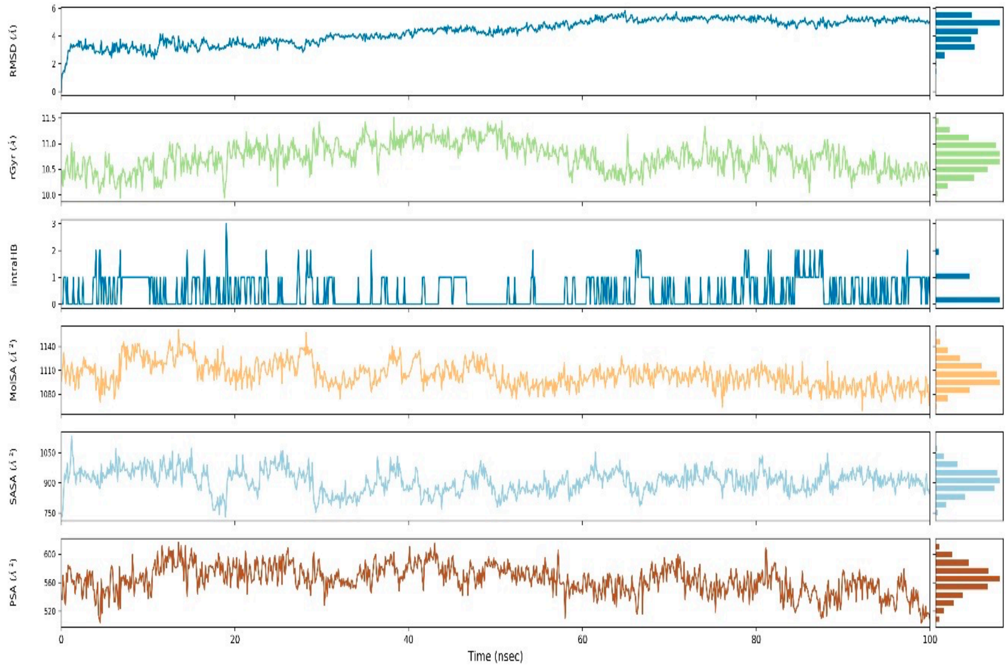

B

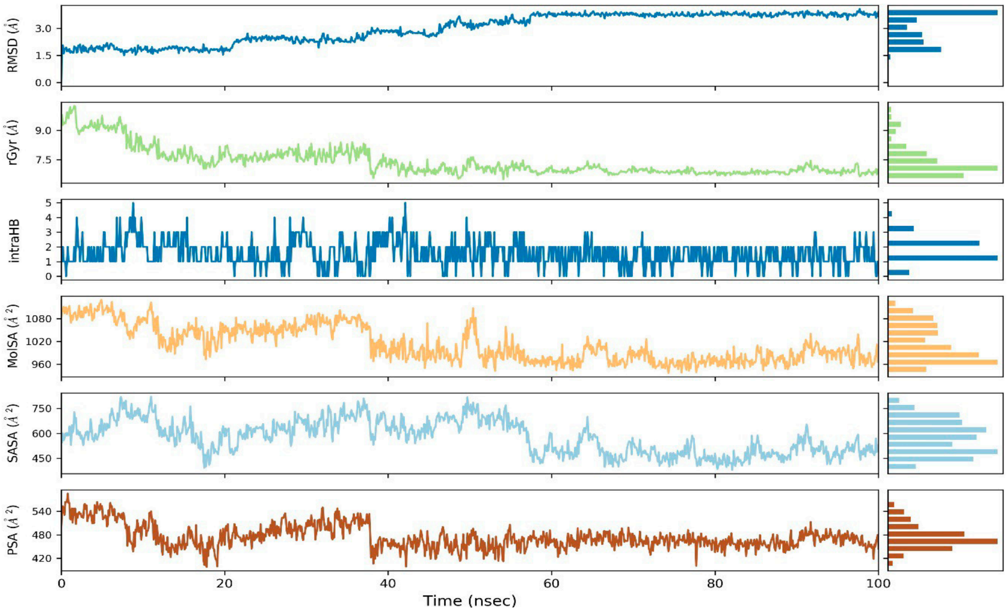

Figure S11. Plots and bar charts of six ligand properties during the simulation (from the bottom): polar surface area (PSA), solvent-accessible surface area (SASA), molecular surface area (MolSA), intramolecular hydrogen bonds (intraHB), radius of gyration (rGyr), and ligand RMSD with respect to the initial conformation. For each property, there is a chart that shows the value of the property as a function of time; to the right, there is a bar chart that shows the proportion of time spent in each of 10 value ranges, divided equally over the range of property values. The subsequent sets of plots and bars are presented for the MOR (**A**) and the D2R (**B**).
